# Reducing the Risk of Secondary Lung Cancer in Treatment Planning of Accelerated Partial Breast Irradiation

**DOI:** 10.3389/fonc.2020.01445

**Published:** 2020-08-18

**Authors:** Nienke Hoekstra, Steven Habraken, Annemarie Swaak-Kragten, Sebastiaan Breedveld, Jean-Philippe Pignol, Mischa Hoogeman

**Affiliations:** ^1^Department of Radiation Oncology, Erasmus MC Cancer Institute, Rotterdam, Netherlands; ^2^Department of Radiotherapy, Dalhousie University, Halifax, NS, Canada

**Keywords:** breast cancer, radiotherapy techniques, accelerated partial breast irradiation, plan optimization, secondary lung cancer

## Abstract

**Purpose:** Adjuvant accelerated partial breast irradiation (APBI) results in low local recurrence risks. However, the survival benefit of adjuvant radiotherapy APBI for low-risk breast cancer might partially be offset by the risk of radiation-induced lung cancer. Reducing the lung dose mitigates this risk, but this could result in higher doses to the ipsilateral breast. Different external beam APBI techniques are equally conformal and homogenous, but the intermediate to low dose distribution differs. Thus, the risk of toxicity is different. The purpose of this study is to quantify the trade-off between secondary lung cancer risk and breast dose in treatment planning and to compare an optimal coplanar and non-coplanar technique.

**Methods:** A total of 440 APBI treatment plans were generated using automated treatment planning for a coplanar VMAT beam-setup and a non-coplanar robotic stereotactic radiotherapy beam-setup. This enabled an unbiased comparison of two times 11 Pareto-optimal plans for 20 patients, gradually shifting priority from maximum lung sparing to maximum ipsilateral breast sparing. The excess absolute risks of developing lung cancer and breast fibrosis were calculated using the Schneider model for lung cancer and the Avanzo model for breast fibrosis.

**Results:** Prioritizing lung sparing reduced the mean lung dose from 2.2 Gy to as low as 0.3 Gy for the non-coplanar technique and from 1.9 Gy to 0.4 Gy for the coplanar technique, corresponding to a 7- and 4-fold median reduction of secondary lung cancer risk, respectively, compared to prioritizing breast sparing. The increase in breast dose resulted in a negligible 0.4% increase in fibrosis risk. The use of non-coplanar beams resulted in lower secondary cancer and fibrosis risks (*p* < 0.001). Lung sparing also reduced the mean heart dose for both techniques.

**Conclusions:** The risk of secondary lung cancer of external beam APBI can be dramatically reduced by prioritizing lung sparing during treatment planning. The associated increase in breast dose did not lead to a relevant increase in fibrosis risk. The use of non-coplanar beams systematically resulted in the lowest risks of secondary lung cancer and fibrosis. Prioritizing lung sparing during treatment planning could increase the overall survival of early-stage breast cancer patients by reducing mortality due to secondary lung cancer and cardiovascular toxicity.

## Introduction

The prognosis of early-stage breast cancer patients is excellent, with a cancer-specific survival of almost 99% at 5 years (1). However, the mortality from radiation induced secondary cancers, especially lung cancers, may offset the survival benefit for certain subgroups (2, 3). There are several models that quantitatively relate dose to the lungs to the risk of secondary lung cancer (4, 5). Thus, reducing the amount of radiation to the lungs during treatment planning could reduce the long-term overall mortality of early stage breast cancer patients.

One option is the use of accelerated partial breast irradiation (APBI) instead of whole breast irradiation (WBI) for early stage breast cancer patients that are eligible according to international guidelines (6–11). Long-term results of randomized trials indicate that local control and survival are non-inferior to whole breast radiotherapy (12–17). Dose comparison studies have shown that the dose to the lungs is significantly lower with APBI compared to WBI but varies greatly depending on the APBI technique used (3, 18–23). The conformality and homogeneity of the different contemporary APBI techniques were similar (21–23). This means that the differences between the external beam APBI techniques are not in the high dose region but in the intermediate and low dose regions where radiation induced malignancies occur. The protocols used in these studies accepted a high lung dose constraint without recommendation to minimize the lung dose well below this constraint. It is unknown to what extent the lung dose can be reduced if highly prioritized during treatment planning and how this impacts the dose to other organs. In the case of APBI, reducing the dose to the lungs mainly results in a higher dose to the ipsilateral non-target breast tissue. For example, this might result in more breast toxicity including fibrosis.

The aim of this study was 2-fold: First, we explored the trade-off between reduction of the mean lung dose as a surrogate of secondary cancer risk and the ipsilateral breast dose distribution as a surrogate of the breast fibrosis risk. Second, we compared coplanar and non-coplanar external beam APBI treatment techniques, using two state-of-the-art techniques, VMAT APBI and stereotactic CyberKnife APBI (CK APBI).

## Materials and Methods

### Patients and CT-Scans

Anonymized CT data of 20 female early-stage breast cancer patients treated at Erasmus MC were included. We randomly selected patients that were previously treated with WBI after breast conserving surgery at our institution. Ethical approval for this retrospective study was not required according to Dutch legislation and the Central Committee on Research Involving Human Subjects. All patients were eligible for APBI according to the 2017 ASTRO selection guidelines (9) and institutional guidelines, meaning they were at least 50 years of age and had a Tis or T1 tumor of <2.5 cm. At Erasmus MC APBI is not used if the ratio of the PTV to the ipsilateral breast volume is more than 30%. A dose of 28.5 Gy in five daily fractions is prescribed.

All patients had a free breathing planning CT-scan in supine position with both arms raised. The tumor bed was delineated as the volume encompassing the seroma, the post-operative changes and the surgical clips. It was expanded with a uniform margin of 10 mm to create the CTV, excluding the thoracic wall and the skin. The skin was defined as the first 5 mm within the patient contour. Accounting for daily image guidance, a CTV to PTV expansion of 5 mm was used.

The delineated organs at risk (OARs) included the ipsilateral and contralateral lungs and breasts, the non-target breast tissue, defined as the ipsilateral breast minus PTV, and heart.

### Treatment Planning

We created coplanar VMAT and non-coplanar CyberKnife stereotactic APBI plans using Erasmus-iCycle (24). For both techniques, the 28.5 Gy isodose line had to encompass at least 95% of the PTV volume. The maximum allowed dose over all voxels was 33 Gy. Planning constraints are summarized in Table 1.

**Table 1:** Planning constraints.

**Structure**	**Clinical constraints**
PTV coverage	28.5 Gy in at least 95%
Ipsilateral breast	V_30Gy_ < 20%, V_15Gy_ < 40%
Contralateral breast	Maximum dose to 2 cc of 1 Gy
Ipsilateral lung	V_9Gy_ < 15%
Contralateral lung	V_1.5Gy_ < 15%
Heart	Right-sided lesions: V_1.5Gy_ < 5% Left-sided lesions: V_1.5Gy_ < 40%

Erasmus-iCycle is an optimizer for multi-criterial beam-profile optimization and optional beam-angle selection applicable to coplanar and non-coplanar IMRT, VMAT, and stereotactic RT. It uses a wish-list, including planning constraints and prioritized objectives. Details and validation of the algorithm have been described elsewhere (24, 25). Plans created by Erasmus-iCycle are Pareto-optimal, which means that it is not possible to improve one objective without deteriorating another one. The primary endpoint of this study was the dose distribution in the intermediate and low dose regions. As these regions are outside the actual target and include different densities such as lung, there may be a loss of electronic equilibrium. A Monte-Carlo based dose engine accurately accounts for these situations. We used the dose engine called GPUMCD (26).

The APBI wish-list for this study included constraints on the maximum dose to the PTV, heart and contralateral breast as well as constraints on conformality. The first objective was to ensure a PTV coverage of at least 95% of the volume with a minimum of 28.5 Gy. The second objective was to minimize the dose to the lungs and the ipsilateral breast. The clinical constraints are detailed in Table 1 and were derived from constraints used in clinical trials on external beam APBI, and more specifically stereotactic APBI using a five fraction regimen (27–29). Left- and right-sided cases were optimized using separate wish-lists with different heart constraints.

Starting from a single personalized plan that equally weighted lung and ipsilateral breast tissue dose, we varied the priorities in 10 incremental steps. This resulted in 11 plans per patient and per technique, ranging from maximally sparing the lung to maximally sparing the ipsilateral breast tissue. During this phase, the dose and coverage of the PTV and the doses to the other OARs were kept constrained to their already obtained values. The prioritization weights varied from a maximum reduction of dose to the breast tissue to a maximum reduction of the lung dose, in nine incremental steps in between. This resulted for each patient in 11 Pareto-optimal plans per technique, covering the full range of lung and breast dose sparing possible.

Coplanar VMAT was planned using 27 coplanar beams with 10° separation to create an arc of 260 degrees. For left-sided cases, the arc ranged from 280 to 180° and for right-sided cases from 80 to 180°. The non-coplanar CyberKnife technique used a multi-leaf collimator and a set of 41 nodes typically used clinically. For both techniques the energy was 6 MV. The planning optimization for the two techniques used the same wish-list, to ensure that trade-offs between PTV coverage, conformality, and organ at risk sparing were identical between the coplanar and non-coplanar plans.

### Analysis

For all plans, we collected an identical set of dose parameters including PTV coverage and mean doses to the lungs, the entire ipsilateral breast, the non-target ipsilateral breast tissue, and the heart.

We calculated the risk of ipsilateral breast fibrosis using the model of Avanzo et al. with complete repair, since the fractionation was once daily (30). The parameters used were BEUD50 = 107.2 Gy, volume parameter *n* = 0.06, slope of dose response *m* = 0.22 and α/β ratio = 3 Gy.

We calculated the risk of secondary lung cancer for all scenarios using the model of Schneider et al. (5). This model calculates the excess absolute risk (EAR) of secondary cancer for an organ at a specified age a and with a radiation exposure at age x. It takes into account the effects of dose fractionation, repair and repopulation and is based on the full dose distribution within an organ. The parameters used were β = 8.0, γe = 0.002, γa = 4.23, agea = 70 years, agex = 50 years, *R* = 0.83, α = 0.042 Gy-1, and α/β = 3 Gy.

We compared coplanar and non-coplanar plans with the Wilcoxon signed rank test. We compared the left-sided and right-sided cases with the non-parametric unrelated samples Mann-Whitney *U*-test. A *p*-value of 0.05 or less was considered significant. All statistical analyses were done in SPSS Statistics version 25.

## Results

Ofthe 20 early stage breast cancer patients included in this study, 11 cases were right-sided and 9 were left-sided and the median volume of the delineated tumor bed was 11.0 cc (range 1.6–51.7 cc). The median volume of the PTV was 92.2 cc (range 58.6–246.9 cc). The median ratio PTV/ipsilateral breast volume was 15.1% (range 7.2–22.3%).

**Figure 1:**
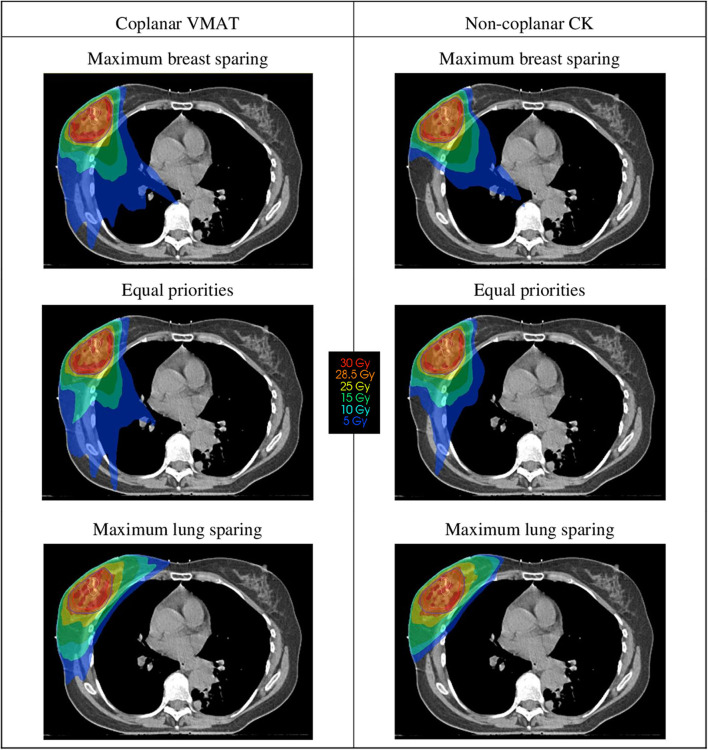
Dose distributions for an example case. The dose distributions on the left show coplanar VMAT plans, on the right non-coplanar CyberKnife (CK) plans. The upper dose distributions are plans with maximum priority to sparing of the breast tissue, and the lower dose distributions are plans with maximum sparing of the lungs. The middle dose distributions are plans with equal priorities to the sparing of lung and breast tissue.

**Figure 2:**
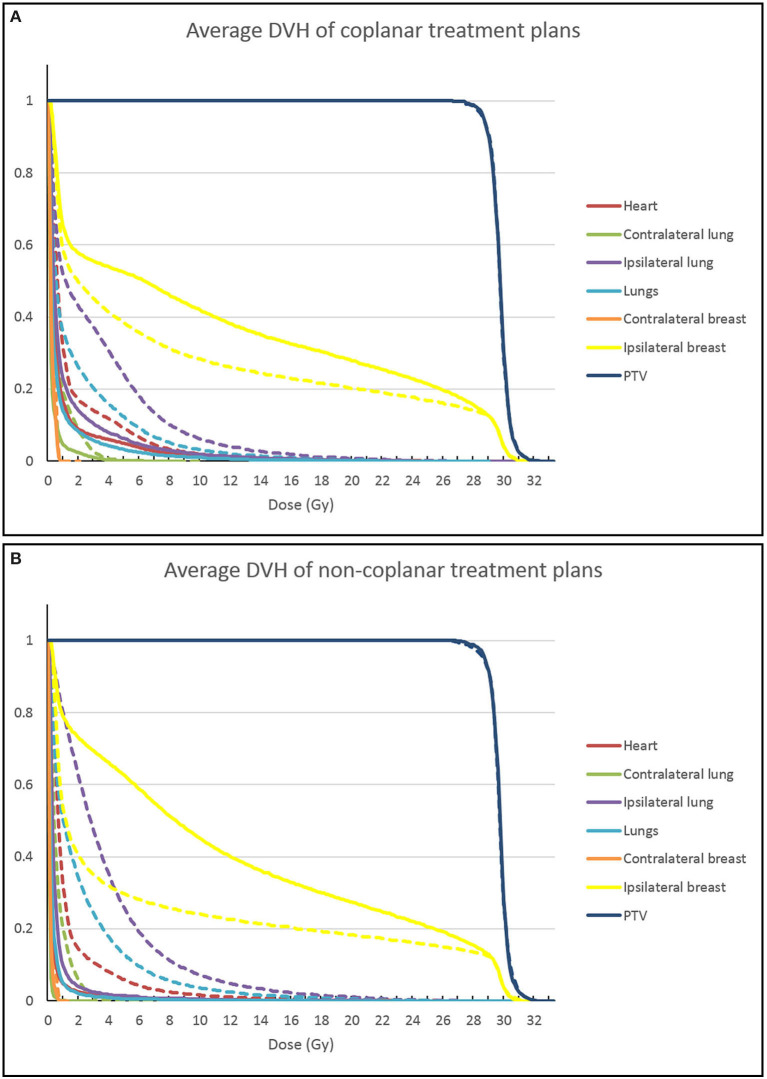
Average DVHs for the different treatment plans. Coplanar VMAT plans are shown in **(A)**, non-coplanar CyberKnife (CK) plans are shown in **(B)**. The solid lines show the results for the plan that fully prioritizes lung sparing. The dashed lines show the result of the treatment plan that fully prioritizes breast sparing.

Figure 1 shows an example of the dose distributions of coplanar and non-coplanar plans with different priorities. When giving more priority to lung sparing, the low dose isodose lines shift from a more opposing configuration to a more tangent one. The average DVHs over 20 patients is shown in Figure 2 for the coplanar and non-coplanar plans for maximum lung sparing and maximum breast sparing.

For 2 out of 20 patients, the coplanar treatment plans did not fulfill the clinical heart constraint. In one left-sided case, the heart was very close to the PTV. The V_1.5Gy_ was 59% with a mean heart dose of 4.6 Gy. The other case was a very medial right-sided tumor. The V_1.5Gy_ for this case was 22% and the mean heart dose 1.6 Gy. The PTV coverage and the other OAR constraints were not violated. Conversely, all non-coplanar plans fulfilled all the constraints.

The dose parameters, secondary lung cancer risks and fibrosis risks are summarized in Table 2. The results for all plans can be found in the Supplementary Files. The median reduction in EAR for secondary lung cancer between the plans with maximum lung sparing and the plans with maximum breast sparing was 5-fold, ranging from 1.1- to 14.8-folds. Comparing VMAT with CK ABPI, the median absolute difference was 11.6 cases per 10,000 patient years for the non-coplanar CK technique and 8.1 cases per 10,000 patient years for the coplanar VMAT technique. The reduction in mean lung dose when prioritizing lung sparing among all patients ranged from 0.21 to 2.06 Gy for the coplanar technique and from 0.69 to 3.37 Gy for the non-coplanar technique. The median reduction of the dose to the ipsilateral breast in the breast sparing plans compared with the lung sparing plans was 3.5 Gy for the coplanar technique (range 0.41–4.73 Gy) and 5.1 Gy for the non-coplanar technique (range 2.21–6.55 Gy). This dose difference resulted in only a very small increase in fibrosis risk of 0.4 and 0.5%, respectively.

**Table 2:** Dose parameters and toxicity risks.

	**Coplanar**			**Non-coplanar**		
	**Breast sparing**	**Equal priorities**	**Lung sparing**	**Breast sparing**	**Equal priorities**	**Lung sparing**
PTV coverage (%)	95.9 (95.6–96.1)	95.9 (95.7–96.0)	96.5 (96.0–96.9)	95.7 (95.4–96.1)	95.5 (95.2–95.9)	96.8 (96.5–97.4)
Lungs mean dose (Gy)	1.9 (1.6–2.2)	1.4 (1.0–1.6)	0.4 (0.3–0.8)	2.2 (2.0–2.5)	1.1 (0.7–1.2)	0.3 (0.2–0.4)
Ipsilateral breast mean dose (Gy)	8.0 (6.8–9.7)	8.4 (7.0–10.1)	11.5 (8.4–12.9)	7.3 (5.4–8.7)	8.2 (6.2–9.4)	12.4 (9.6–13.7)
Non-target breast tissue mean dose (Gy)	4.8 (4.1–5.7)	5.1 (4.4–6.2)	8.3 (6.2–9.4)	3.7 (2.6–4.5)	4.4 (3.5–5.5)	9.8 (7.5–10.5)
Heart mean dose (Gy)						
Left-sided cases	2.3 (1.7–3.3)	1.8 (1.2–3.3)	0.6 (0.2–2.6)	2.0 (1.5–3.0)	1.2 (0.5–2.8)	0.3 (0.1–1.3)
Right-sided cases	0.6 (0.6–0.7)	0.6 (0.5–0.6)	0.4 (0.2–0.5)	0.7 (0.6–0.7)	0.5 (0.5–0.7)	0.1 (0.1–0.2)
EAR secondary lung cancer	11.3 (9.7–13.3)	8.8 (6.9–9.9)	3.2 (2.4–5.3)	13.7 (12.7–15.1)	7.2 (5.6–8.4)	2.1 (1.5–2.6)
Breast fibrosis risk (%)	7.7 (6.3–8.6)	7.7 (6.3–8.6)	8.2 (6.6–9.1)	7.6 (6.2–8.6)	7.5 (6.2–8.5)	8.0 (6.5–8.9)

*All data is shown as median (interquartile range). EAR Excess Absolute Risk per 10,000 patient years*.

**Figure 3:**
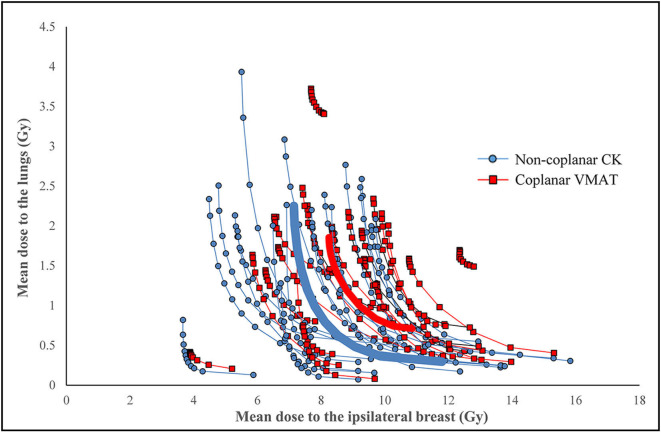
Pareto fronts for individual patients of the mean doses to the ipsilateral breast and lungs. Non-coplanar CyberKnife (CK) plans are shown in blue circles, coplanar VMAT plans are shown in red squares. The thick lines show the average over all patients per technique.

Figure 3 shows the Pareto-fronts of all patients of the trade-off between mean lung dose and mean ipsilateral breast dose. The non-coplanar plans systematically resulted in lower doses to both lungs and ipsilateral breast tissue (Table 3, Wilcoxon signed rank test *p* < 0.001). The Pareto-fronts did not cross for any patient, meaning that for a given dose to one organ, the dose to the other organ was higher in the coplanar treatment plan in all cases.

**Table 3:** Comparison of coplanar and non-coplanar techniques.

	**Coplanar**	**Non-coplanar**	**Wilcoxon signed rank test**
PTV coverage (%)	95.9	95.6	*p* < 0.001
Lungs mean dose (Gy)	1.3	0.9	*p* < 0.001
Ipsilateral breast mean dose (Gy)	9.0	8.3	*p* < 0.001
Non-target breast tissue mean dose (Gy)	5.6	4.7	*p* < 0.001
Heart mean dose (Gy)	0.65	0.63	*p* < 0.001
EAR secondary lung cancer	8.3	6.3	*p* < 0.001
Breast fibrosis risk (%)	8.0	7.7	*p* < 0.001

*Median values over all plans for all cases per technique. EAR Excess Absolute Risk per 10,000 patient years*.

In Table 4, the dose parameters are reported for the left-sided and right-sided case separately. Using the unrelated samples Mann-Whitney *U*-test, there were no significant differences between the two groups except for the mean heart dose. The mean heart dose was higher for the left-sided cases for both techniques and for both the lung sparing plan and the breast sparing plan.

**Table 4:** Dose parameters and toxicity risks for left-sided and right-sided tumor location.

	**Coplanar**				**Non-coplanar**			
	**Breast sparing**		**Lung sparing**		**Breast sparing**		**Lung sparing**	
	**Left-sided**	**Right-sided**	**Left-sided**	**Right-sided**	**Left-sided**	**Right-sided**	**Left-sided**	**Right-sided**
PTV coverage (%)	95.9 (95.8–96.5)	95.9 (95.6–96.1)	96.7 (96.3–97.3)	96.1 (95.9–96.9)	95.6 (95.4–96.3)	95.7 (95.4–95.9)	97.2 (96.4–97.6)	96.8 (96.6–96.9)
Lungs mean dose (Gy)	1.8 (1.5–2.1)	1.9 (1.6–2.3)	0.4 (0.3–1.1)	0.5 (0.4–0.8)	2.2 (2.0–2.5)	2.2 (2.0–2.6)	0.3 (0.2–0.4)	0.3 (0.2–0.3)
Ipsilateral breast mean dose (Gy)	8.3 (6.2–9.9)	7.7 (7.4–9.3)	11.8 (8.3–12.9)	11.2 (9.7–12.9)	7.7 (5.1–9.0)	6.8 (5.5–8.4)	12.4 (9.2–13.9)	12.4 (9.7–13.7)
Non-target breast tissue mean dose (Gy)	4.1 (3.0–6.3)	4.9 (4.5–5.6)	8.2 (4.8–9.7)	8.3 (7.2–9.4)	3.5 (2.0–4.7)	3.8 (2.9–4.4)	8.9 (6.0–10.6)	9.9 (7.7–10.5)
Heart mean dose (Gy)	2.3 (1.7–3.3)	0.6 (0.6–0.7)	0.6 (0.2–2.6)	0.4 (0.2–0.5)	2.0 (1.5–3.0)	0.7 (0.6–0.7)	0.3 (0.1–1.3)	0.1 (0.1–0.2)
EAR secondary lung cancer	10.8 (9.5–12.5)	11.8 (9.6–14.3)	2.9 (2.1–6.3)	3.5 (2.7–5.5)	13.8 (12.7–15.0)	13.5 (12.6–16.1)	2.1 (1.4–3.0)	2.1 (1.7–2.2)
Breast fibrosis risk (%)	8.5 (6.0–9.0)	6.9 (6.3–8.5)	8.7 (6.2–9.2)	7.1 (6.8–8.9)	8.4 (5.8–8.8)	6.8 (6.2–8.4)	8.7 (6.1–9.2)	7.1 (6.5–8.8)

*All data is shown as median (interquartile range). EAR Excess Absolute Risk per 10,000 patient years*.

**Figure 4:**
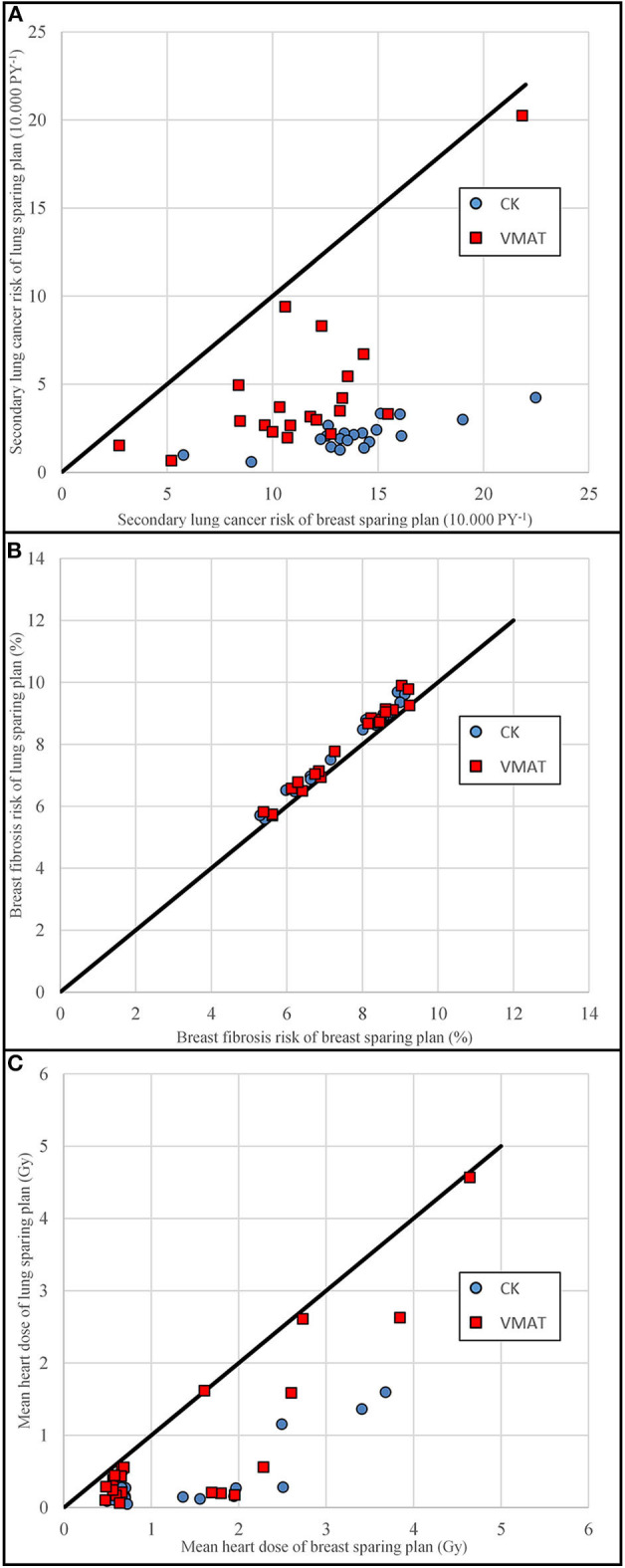
Scatterplots of the maximum breast sparing plans vs. the maximum lung sparing plans. **(A)** shows the secondary lung cancer risk, **(B)** the breast fibrosis risk, and **(C)** the mean heart dose. Non-coplanar CyberKnife (CK) plans are shown in blue circles, coplanar VMAT plans are shown in red squares. Data points below the unity line indicate an advantage for the dose plan on the vertical axis.

Figure 4 details the comparison of the breast sparing plan on the x-axis and the lung sparing plan on the y-axis per individual patient. A point below the unity line means that the lung sparing plan had a lower value than the breast sparing plan. The coplanar plans are shown as circles and the non-coplanar plans as squares. Figure 4A shows that the absolute secondary lung cancer risks were lower for the non-coplanar technique than for the coplanar technique. The fact that the squares are at a larger distance from the unity line suggests that the differences between the lung sparing and breast sparing plans were larger for the non-coplanar technique. The breast fibrosis risks (Figure 4B) are close to the unity line for both techniques.

The dose to the heart was also reduced in the lung sparing plans without any specific constraint to do so. The difference in mean heart dose for the left-sided cases was 1.7 and 1.2 Gy for the non-coplanar and coplanar techniques, respectively. For the right-sided cases, the difference in mean heart dose was 0.5 and 0.2 Gy, respectively. The comparison is shown in Figure 4C.

## Discussion

This study shows that it is possible to dramatically reduce the mean lung dose and hence the risk of secondary lung cancer for APBI by giving a higher priority to lung sparing. The median risk reduction was 5-fold, with a range of 1.1–14.8-folds. This translates into a median absolute risk reduction of 11.6 cases per 10,000 patient years for the non-coplanar technique and 8.1 for the coplanar technique, which is highly clinically relevant, as these patients are expected to survive several decades. The mortality of lung cancer is about 80% (31). Multiplying the absolute risk reduction by 0.8 shows that minimizing the lung dose could theoretically reduce the overall mortality of early-stage breast cancer patients with 9.3 persons per 10,000 patient years for the non-coplanar technique and with 6.5 persons per 10,000 patient years for the coplanar technique.

Shifting the dose away from the lungs resulted in a higher dose to the ipsilateral non-target breast tissue. However, the increase in mean breast dose of 3.5 and 5.1 Gy translated into a small increase in the risk of breast fibrosis of 0.5 and 0.4%-point for the coplanar and non-coplanar technique, which is not clinically relevant. The limited increase in the calculated fibrosis risk could be explained by the NTCP model used, and notably the n parameter of 0.06 used in the model of Avanzo et al. (30). This value means that the risk of fibrosis is defined primarily by the high dose volume. The planning constraints that affect the high dose volume are the PTV constraints. These constraints were kept constant for the plans with different priorities in our study. This resulted in plans with very small differences in the dose to the PTV and consequently in the risk of fibrosis. The differences between the plans with the different priorities were in the intermediate and low dose regions, and these regions have a very limited effect in the fibrosis model.

The Avanzo model for breast fibrosis is the only published model addressing APBI. One weakness of this model is the limited data on which it is based as well as the lack of external validation. However, other models of fibrosis for WBI also found that this risk mainly depends on the high dose region (32, 33). Using the WBI fibrosis model of Mukesh et al. resulted in absolute fibrosis risks of about 17%, but the differences between the breast sparing plans and the lung sparing plans remained very small (32).

An important finding of this study is that the use of non-coplanar beams always resulted in a more favorable dose distribution, as shown in Figure 2. In this study we used CyberKnife stereotactic radiotherapy, which provides more degrees of freedom in treatment planning than coplanar VMAT. The Pareto fronts of the two techniques did not cross for any patient, and the non-coplanar technique always had the lowest doses and lowest risks of secondary lung cancer and breast fibrosis. The non-coplanar technique also had a wider front, showing a larger dynamic range for sparing of specific organs. The difference between the techniques was statistically significant, as is shown in Table 3. The PTV coverage was slightly closer to 95% for the non-coplanar technique. The aim of our automated planning technique was to get a coverage of 95%, but not higher. This means that a coverage closer to 95% is in fact a better result than a higher coverage. The non-coplanar technique has more freedom to get a coverage that is close to the requested value and to reduce doses to other organs.

Incidentally, we found that optimizing lung sparing also resulted in lower doses to the heart. The mean heart dose in the lung sparing plans for the left-sided cases was on average 1.7 and 1.2 Gy lower than in the breast sparing plan for the non-coplanar and coplanar techniques, respectively. This is clinically significant following the model published by Darby et al. who reported a linear dose-response relationship between mean heart dose and cardiovascular events without a threshold. This means that optimizing lung sparing would also result in a lower risk of cardiovascular events (34–36).

There are some limitations in the present study. We did not compare all possible external beam APBI techniques. For example, 3D-conformal APBI is often used, but we have chosen to use only the coplanar and non-coplanar techniques with the highest conformality. 3D-conformal APBI would have resulted in an artificially increased dose to the ipsilateral non-target breast tissue. Intuitively there are concerns that the lung dose might be higher with VMAT compared to 3D-conformal RT. However, Essers et al. showed that the lung dose would be lower with VMAT when using partial arcs (37). In our study, the optimizer was free to select from all available beam angles, and it chose only partial arcs for the lung sparing plans and not for the breast sparing plans. This is in agreement with the conclusion of Essers et al.

We have chosen to use Erasmus-iCycle because it can generate true bias-free comparisons using exactly the same wish-list for all plans without any human interference. Treatment plans created in Erasmus-iCycle are based on fluence map optimization. They need to be converted into segmented dose plans before the plan is deliverable to a real patient. With the right algorithm, the difference between fluence map optimized plans and segmented plans is small (38). The VMAT and CK would need to be converted using different treatment planning software with different dose calculation algorithms. This could influence the results. The dose parameters for our plans with equal priorities are comparable to the dose levels reported in literature (23).

We used the full model of Schneider et al. for the calculation of the secondary lung cancer risk (5). This model takes into account cell killing and fractionation effects and uses the full dose distribution in the organ. It is specifically created for radiotherapy patients, but it is based on limited epidemiological data. The BEIR VII model is based on more extensive epidemiological data, but this model is made for radiation protection purposes and intended for use in low dose exposures only (4). Using this model would result in the same conclusion, that prioritizing lung sparing reduces the secondary cancer risk. For this model, the reduction for non-coplanar CK is 8.9-fold and for coplanar VMAT 3.9-fold, compared to 6.5- and 3.5-fold, respectively for the Schneider model.

In conclusion, the risk of secondary lung cancer of external beam APBI can be greatly reduced by prioritizing lung sparing during treatment planning. The associated increase in breast dose did not lead to a relevant increase in fibrosis risk. Lung sparing also resulted in a lower mean heart dose. Thus, prioritizing lung sparing could increase the overall survival of early-stage breast cancer patients by reducing mortality due to secondary lung cancer and cardiovascular toxicity. The use of non-coplanar beams resulted in both a lower secondary lung cancer risk and a lower fibrosis risk which suggests that it should be favored for breast APBI.

## Data Availability Statement

All datasets generated for this study are included in the article/Supplementary Material.

## Ethics Statement

Ethical approval for this retrospective study was not required according to Dutch legislation and the Central Committee on Research Involving Human Subjects.

## Author Contributions

NH, SH, J-PP, and MH contributed conception and design of the study. NH performed the statistical analysis and wrote the first draft of the manuscript. All authors contributed to manuscript revision, read, and approved the submitted version.

## Conflict of Interest

NH reports grants from Accuray Inc., Sunnyvale, USA, during the conduct of the study. MH has been member of the Clinical Advisory Board of Accuray Inc., Sunnyvale, USA. The remaining authors declare that the research was conducted in the absence of any commercial or financial relationships that could be construed as a potential conflict of interest. Erasmus MC Cancer Institute has research collaborations with Accuray Inc., Sunnyvale, USA, and Elekta AB, Stockholm, Sweden.
